# Metastatic Papillary Thyroid Carcinoma in a Paediatric Patient

**DOI:** 10.1155/2021/6655491

**Published:** 2021-01-05

**Authors:** Nthabiseng Ellen Mothata, Takalani Gidion Morulana, Nyaweleni Tshifularo, Phumudzo Bridgett Nemutaduni, Nozipho Elizabeth Nyakale, Moshawa Calvin Khaba

**Affiliations:** ^1^Department of Anatomical Pathology, Dr George Mukhari Academic Laboratory, National Health Laboratory Services, Sefako Makgatho Health Sciences University, Ga-Rankuwa, South Africa; ^2^Department of Paediatric Surgery, Dr George Mukhari Academic Hospital, Sefako Makgatho Health Sciences University, Ga-Rankuwa, South Africa; ^3^Department of Nuclear Medicine, Dr George Mukhari Academic Hospital, Sefako Makgatho Health Sciences University, Ga-Rankuwa, South Africa

## Abstract

Papillary thyroid carcinoma is the most common endocrine cancer in the paediatric population. Although the disease is diagnosed at a later stage, the prognosis is favourable. When these patients present with lymph nodal and/or pulmonary metastases, they may be initially confused for infectious diseases such as tuberculosis. Therefore, thorough clinical assessment including radiology and microbiological and histopathological assessment is important for early and correct diagnosis. We report an 11-year-old female patient who presented with cervical lymphadenopathy and the histopathological assessment confirmed metastatic papillary thyroid carcinoma. Subsequent radiological investigation revealed further metastasis to the lung. This manuscript highlights the difficulties that might be encountered in the initial management of paediatric PTC which present atypically.

## 1. Introduction

Paediatric thyroid carcinomas are rare endocrine cancers, majority of which are comprised of papillary thyroid carcinoma (PTC) followed by follicular carcinoma [1]. They are associated with radiation exposure, thyroid disease such as Hashimoto's thyroiditis, and inherited condition such as familial adenomatous polyposis. They are more common in older children and adolescence with a female-to-male ratio of 5 : 1 [2, 3]. Approximately less than 3% of the patients have thyroid nodules which should raise a high index of suspicion for malignancy [1, 4]. A definite diagnosis is usually delayed due to the subtle presentation of the primary malignancy, and therefore, they may present late with metastatic disease [1]. The gold standard of diagnosis is histopathology. Surgery and radioactive iodine are the treatment of choice in these patients and are dependent on the risk groups [5]. Although they present with metastatic disease, the prognosis still remains good [2]. We present a paediatric patient who presented atypically with metastatic PTC to the cervical lymph nodes and lungs.

## 2. Case Presentation

**Figure 1:**
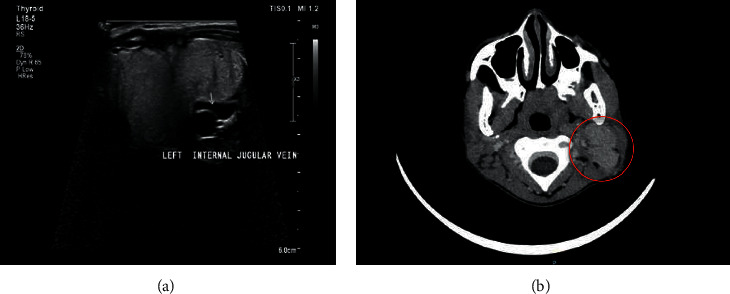
(a) Neck ultrasound shows enlarged left cervical lymph node compressing on the left internal jugular vein; (b) CT scan shows mattered left cervical lymphadenopathy (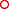).

An 11-year-old African female patient presented to paediatric surgery department with a 4 months' history of lump on the left side of the neck. She gave a history of a painless and slow-growing lump on the left side of the neck that was treated with antibiotics by several general practitioners with no improvement. She did not have any comorbidities; however, her mother has a thyroidectomy for nodular goiter. She appeared chronically ill without pallor, jaundice, or proptosis. Her vital signs were within normal limits. She had a lateral neck mass that measured 50 mm × 40 mm which was mobile, nontender, and hard with attachment to the skin. Other systemic examinations were unremarkable. Blood investigation was within normal limits including thyroid function tests. A neck ultrasound showed an enlarged left cervical lymph node with a normal blood flow in the right and left thyroid lobes. There was a macrolobulated lesion (TIRADS 5) with increased flow and mass effect to the internal jugular vein (Figure 1(a)). A lymph node biopsy was done and confirmed a metastatic papillary thyroid carcinoma. A subsequent staging computed tomography (CT) scan showed a distinct focal peripheral mass lesion on the inferior pole of the left thyroid lobe with calcifications, mass effect on the left internal jugular vein (Figure 1(b)), and bilateral upper lung lobe nodules. An uneventful total thyroidectomy with a modified neck dissection was done and sent for histopathological assessment.

Gross pathology showed a thyroid tumour that measured 55 × 35 × 10 mm and matted lymph nodes. The tumour was unencapsulated, hemorrhagic, and firm without necrosis.

**Figure 2:**
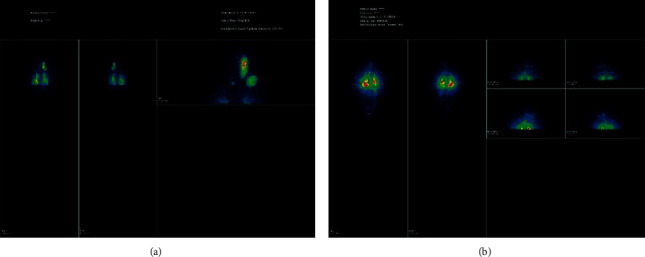
(a) I^123^ and (b) I^131^ show increased nonuniform uptake in level II, III, and IV cervical lymph nodes, mild residual uptake in the thyroid bed, and increased uptake in the lungs bilaterally.

Microscopic examination confirmed papillary variant of PTC evidenced by tumour arranged in papillae, nests, and follicles (Figures 2(a) and 2(b)). These structures were lined by follicular cells with nuclei exhibiting grooves, clearing, pseudoinclusions, overlapping, and crowding. In addition, five lymph nodes that were sampled showed metastatic disease.

**Figure 3:**
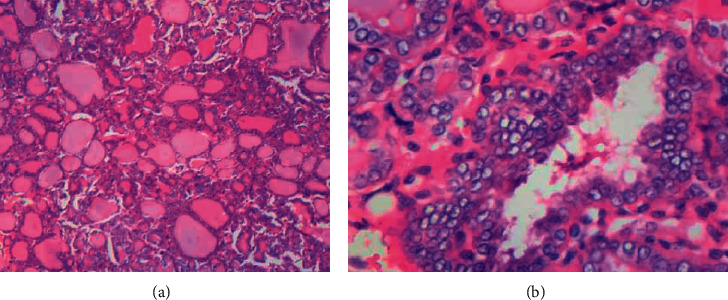
(a) Thyroid tumour with papillary and follicular pattern; (b) typical nuclear features of PTC: nuclear overlapping, clearing, and occasional grooves.

An iodine-123 single photon emission computed tomography (SPECT) scan done postsurgery, under high thyrotropin stimulation (>30 mU/L), showed increased nonuniform uptake in level II, III, and IV cervical lymph nodes, mild residual uptake in the thyroid bed, and increased uptake in the lungs bilaterally (Figure 3(a)). These features confirmed metastatic disease to the lymph nodes and lungs.

A left lateral lymph node clearance was done which further confirmed metastatic papillary thyroid carcinoma. Four weeks postsurgery, the patient received 100 mCi of I-131 therapy. This was under TSH stimulation with the withdrawal of eltroxin for 4 weeks and given I^131^ under maximally stimulated TSH. Prophylactic dose of prednisone was given in conjunction with this therapy to minimize inflammatory response that may result from the radiation.

The posttherapeutic iodine-131 images (Figure 3(b)) done prior to discharge were congruent in pattern to the staging iodine-123 scan images. Thyroid function tests were low (thyroxine: 10.4 pmol/L, tri-iodo thyronine: 5.6 pmol/L, and thyroid-stimulating hormone: 0.54 mIU/L). The patient was discharged on eltroxin daily.

At the 6^th^ month follow-up visit, she tolerated the RAI well with improved nutritional status. She was euthyroid with TSH: 4.13 mIU/L, T4: 9.4 pmol/L, T3: 6.0 pmol/L, and thyroglobulin: >300 *μ*g/L. She was put on lifetime thyroid hormone replacement therapy.

## 3. Discussion

Papillary thyroid carcinoma is the leading cause of paediatric endocrine cancers with an incidence of 6% of all cancers [1]. In typical cases of PTC, patients present as multifocal involving both lobes and 50% of children present with cervical lymph node metastasis and lung metastasis. The initial evaluation of thyroid nodule or neck mass is serum TSH and T4 which in most cases is normal at presentation, followed by neck ultrasound. This is followed by a confirmatory fine-needle aspiration (FNA) [6]. Our patient presented with atypical feature, an asymptomatic neck mass on the left side of the neck; therefore, a normal workup for thyroid disease was not followed. She did not have risk factors for thyroid malignancy; hence, a clinical diagnosis of thyroid malignancy was not entertained. Whilst Hashimoto's thyroiditis (HT) is the commonest risk factor associated with paediatric PTC [7], the patient did not report any history of autoimmune disease. Furthermore, histopathological assessment of thyroid did not reveal background HT. While thyroid function tests would not have helped as they are usually normal, FNA of the mass should still be done. This helps to exclude nonmalignant and malignant disease. Clinician should always have a high index of suspicion for malignancy in children who presents with asymptomatic neck mass.

The histomorphological features of PTC in children are the same as in adults. Although it may present with varied growth patterns including papillary, follicular, and solid, amongst others, the diagnosis is based on typical nuclear features. These features include Orphan Annie eye nuclei which are optically clear. They also exhibit pseudoinclusion and grooves [8]. The diagnosis on the index patient was done based on these features.

The management of PTC involves extensive planning from staging preoperatively and deciding on approach that will benefit both the patient and family. Given the very high rate of metastatic disease in this population, preoperative neck ultrasound to identify suspicious lymph nodes and chest X-ray and/or chest CT should be considered in these patients as it will guide on the type of surgical treatment to be followed.

The latest American Thyroid Association (ATA) children's guidelines, published in 2015, recommend radioactive iodine therapy (RAI) after total thyroidectomy for intermediate- and high-risk groups who have extensive central neck lymph node involvement or locally invasive or extensive lateral neck cervical lymph nodes. The aim of RAI is to increase the response rate to surgical management. It has been reported that when radioactive iodine therapy is used in conjunction with thyroid-stimulating hormone (TSH) suppression, the rate for complete response is 47.32% and for partial response is 38.39%. Postsurgical RAI therapy is indicated in paediatric PTC due to its increased risk of bilateral (30%) or multifocal involvement (65%) [6, 8].

Lymph node dissection is recommended to reduce the risk of recurrence and increase the efficacy of RAI therapy in patients with extrathyroidal invasion or locoregional metastasis [8–10].

The index patient was in the high-risk group, and hence, a total thyroidectomy with radical neck dissection and radioactive therapy was the preferred choice of treatment.

The follow-up guidelines include serum thyroglobulin on levothyroxine every 3–6 months for 2–3 years then annually and thyroid ultrasound including cervical lymph nodes for 6–12 months for intermediate- and high-risk groups and annually for low-risk patients. In case the thyroglobulin level is elevated, and the thyroid ultrasound is negative, then a CT scan of the chest and neck is recommended. In children, more than 98% of cases have a good survival rate at 10 years while few patients develop progressive or refractory disease not amenable to surgical intervention or resistant to radioactive therapy.

The prognosis for PTC is excellent with low mortality rate. The survival rate for different risk groups is as follows: 99% for low-risk groups, 83% for intermediate-risk groups, and 43% for high-risk groups [11].

The survivors of childhood ductal thyroid carcinoma need a follow-up for life since recurrence has been reported at 40 years after initial therapy [8].

In conclusion, fine-needle aspiration may provide early diagnosis in children with neck mass and avoid delayed management. A high index of suspicion for PTC should be maintained, regardless of risk factors. This allows for early targeted treatment with reduction in morbidity and mortality in this population. Surgical treatment with or without lymph node dissection and radiotherapy remains the cornerstone of management in paediatric PTC.

## Data Availability

The relevant data and materials are available from the corresponding author upon request.
